# Interaction Between Functionally Activate Endometrial Microbiota and Host Gene Regulation in Endometrial Cancer

**DOI:** 10.3389/fcell.2021.727286

**Published:** 2021-09-23

**Authors:** Peigen Chen, Yingchun Guo, Lei Jia, Jing Wan, TianTian He, Cong Fang, Tingting Li

**Affiliations:** ^1^Reproductive Medicine Center, The Sixth Affiliated Hospital, Sun Yat-sen University, Guangzhou, China; ^2^The Third Affiliated Hospital, Sun Yat-sen University, Guangzhou, China

**Keywords:** endometrial cancer, host–microbe interactions, gene regulation, microbiome, tumor migration

## Abstract

**Objective:** In this study, we mainly explored two questions: Which microorganisms were functionally active in the endometrium of patients with endometrial cancer (EC)? What kind of response did the human host respond to functionally active microorganisms?

**Methods:** Nine endometrial cancer patients and eight normal subjects were included in this study. HMP Unified Metabolic Analysis Network 3 (HUMAnN3) was used to obtain functional information of microorganisms. In addition, metaCyc-based GSEA functional enrichment analysis was used to obtain information on the metabolic pathways of the human host. At the same time, the O2PLS model and Spearman correlation analysis were used to analyze the microorganisms–host interaction.

**Results:** With the novel metatranscriptome analysis pipeline, we described the composition of more than 5,000 functionally active microorganisms and analyzed the difference in microorganisms between the EC and the normal group. Our research found that these microorganisms were involved in part of the metabolic process of endometrial cancer, such as 6-sulfo-sialyl Lewis x epitope, *N*-acetyl-beta-glucosaminyl. In addition, the host–microbiota crosstalk of EC endometrium also included many biological processes, mainly functions related to tumor migration and the Apelin signaling pathway.

**Conclusion:** The functionally active microorganisms in the EC endometrium played an essential role in the occurrence and migration of tumors. This meant that functionally active microorganisms could not be ignored in the treatment of endometrial cancer. This study helped to better understand the possible role of endometrial functional, active microorganisms in the occurrence and development of EC in patients with endometrial cancer and provided new information for new attempts to treat EC.

## Introduction

Endometrial cancer (EC) is the fourth most common malignant tumor in women (Miller et al., 2020). The number of new endometrial cancers worldwide is about 382,100 per year, and nearly 89,900 patients die from endometrial cancer each year (Ferlay et al., 2019). In addition, there are nearly 64,000 new cases in China every year, resulting in 16,000 deaths (Chen W. et al., 2018). Moreover, this number was still rising, which was a considerable challenge.

For an extended period, the uterus was considered sterile. With the development of detection technology, the endometrium was proved to have its resident microbiota, the microbial community (Koedooder et al., 2019). The microbiota was closely related to the occurrence and development of cancer. For example, it could cause cancer by inhibiting cell apoptosis, stimulating proliferation, and interfering with genome stability (Baker et al., 2018). A study based on 16S sequencing technology by Walther-Antonio et al. (2016) showed that endometrial cancer patients and endometrial hyperplasia patients had changes in the microbiota structure of the endometrium, and there was a significant difference in the structure of the endometrial microbiota in mild cases. Another study by Lu et al. (2021) suggested that the endometrial microbiota were closely associated with the disorder of inflammatory cytokines in EC. These studies confirmed that the endometrial microbiota might play an essential role in EC. However, the host’s response mechanism had not yet been elucidated.

In addition, the current standard methods of microbial research, such as 16s rRNA and metagenome analyses, were all based on DNA sequences. However, the existence of characteristic DNA sequences was not equal to the existence of living microorganisms. Furthermore, since DNA molecules might exist for decades (Glassing et al., 2016), the DNA sequence might originate from the decomposition of microorganisms, such as DNA from dead microorganisms (Aagaard et al., 2014). Therefore, research methods based on DNA sequence could not confirm the existence of microbiomes (Glassing et al., 2016; de Goffau et al., 2018; Sola-Leyva et al., 2021).

In this study, based on the functional, active endometrial microbiota mapping pipeline using metatranscriptome (meta-RNA sequencing analysis) (Macklaim and Gloor, 2018; Liu et al., 2020; Sola-Leyva et al., 2021), we explored the changes and activity of endometrial microbiota in EC. Metatranscriptomics could analyze microbial transcript profiles using RNA-seq data to identify living microorganisms and their functions (Macklaim and Gloor, 2018; Liu et al., 2020). At the same time, we combined with the host endometrial transcriptome to analyze the effects of the microbiota on the endometrium or the host’s response to changes in the microbiota.

## Materials and Methods

### Study Material

The data of nine endometrial cancer patients and eight control subjects were included in this study. The raw SRA files of RNA-seq were extracted from PRJNA612305 from the Gene Expression Omnibus (GEO) repository. As mentioned earlier (DiGuardo et al., 2021), the Qubit RNA BR assay (Invitrogen, Carlsbad, CA, United States) was used to quantify total RNA, and the DV200 value (percentage of RNA fragments >200 bases in length) was used to assess the quality using the 2100 Bioanalyzer RNA Nano kit (Agilent Technologies, Santa Clara, CA, United States). The clinical data of the subject cohort included in this study were: EC cohort: *n* = 9, age: 68.2 ± 10.3 years (mean ± standard deviation) and CON cohort: *n* = 8, age: 61.8 ± 13.5 years.

### Functionally Active Endometrial Microbiota Mapping

First, the raw SRA files were converted into FASTQ format by using SRA toolkit (parameter –split-files) (Staff, 2011). Then, these FASTQ files were processed by using fastp (Chen S. et al., 2018) to remove reads containing adapters, more than 10% unknown nucleotides (N), and more than 50% of low-quality (*Q*-value ≤ 20) bases. Subsequently, the remaining sequences were aligned to the human reference genome GRCh38 from Gencode v26 using HISAT2 (Kim et al., 2015). The comparison results were stored in a separate .sam file. At the same time, the sequence (non-human sequence) that cannot be aligned with the human reference genome was stored as a separate FASTQ file (parameter –un-conc-gz). Next, the Kraken2 reference database was used to align these non-human sequences and output in mpa style (parameter –use-mpa-style) (Wood et al., 2019). Next, the reference database (including Bacteria, Archaea, and Viruses library) was downloaded using the Kraken-build utility. The scope of the database included the classification information of the National Center for Biotechnology Information (NCBI) and the complete genome sequence of RefSeq. Then metagenomeSeq R package (Paulson et al., 2013) was used to identify the different species in microorganismal communities of EC and normal (control) group with the directly assigned read counts. First, the results of the Kraken2 comparison were further carried out to remove rare species (at least in four samples, the count was greater than one) and normalized by using the Cumulative Sum Scale (CSS) method (Paulson et al., 2013). The process of the CSS algorithm was mainly to obtain a percentile by dividing the raw count by the cumulative sum of the counts. In this way, the relatively constant count distribution in the dataset was captured to process the raw count. The advantage of this treatment was that compared to ratio-based normalization or random sampling methods, CSS had a higher sensitivity (Paulson et al., 2013). Subsequently, the normalized data were used for differential species abundance analysis, using a zero-inflated log-normal model in metagenomeSeq.

### Functional Enrichment Analysis of Endometrium Microbial Metabolic Pathways

The HMP Unified Metabolic Analysis Network 3 (HUMAnN3) (Franzosa et al., 2018) was used to describe the metabolic potential of members of a microbial population based on the MetaCyc database. Then we used the HUMAnN_renorm_table script to normalize the data for count per million (CPM). Finally, all the results were merged into one file through the HUMAnN_join_tables utility. Next, different metabolic pathways were screened by STAMP software (version 2.1.3) (Parks et al., 2014).

### Host Transcriptome Analysis

Samtools (Li et al., 2009) was first used to transfer the sam file (as mentioned before, aligned to the human reference genome) to bam file. We then used featureCounts (Liao et al., 2014) to quantify the gene expression value. Based on the raw count calculated by featureCounts, DEseq2 R package (Love et al., 2014) was used to select the differential expression genes between EC and normal group with log2-fold-change ≥ | 1| and adj *p*-value < 0.05. Since microbial metabolic pathways were analyzed based on the MetaCyc database, we used the Pathway Tools utility to obtain all human metabolic pathways (HUMAN2cyc, version: 24.5) in the MetaCyc database. The obtained pathway .col file contains a total of 390 human metabolic pathways. Then we converted the .col file into a gene set enrichment analysis (GSEA) input file in GMT format and used the GSEAPreranked method in GSEA to perform enrichment analysis of human metabolic pathways based on the results of the difference analysis (Subramanian et al., 2005).

### Comprehensive Analysis of Interactions Between Endometrial Microbial Community Changes and Host Gene Dysregulations

The Two-way Orthogonal PLS (O2PLS) model (Trygg and Wold, 2003) showed excellent performance in the integrated analysis of multi-omics data, and its estimated value was close to the actual parameters in both low-dimensional and high-dimensional data (Bouhaddani et al., 2016). This study used the O2PLS model to conduct a comprehensive analysis of endometrial microbial community changes and host gene dysregulations using OmicsPLS R package (Bouhaddani et al., 2018). First, we used the crossval_o2m_adjR2() function to determine the best parameters through sevenfold cross-validation. Then o2m() function was used for modeling. Next, ClusterProfiler R package (Yu et al., 2012) was performed for functional enrichment analysis of the top 50 genes loading.

In order to analyze the activities of each different species, Spearman correlation analysis was used to explore the relationship between species and species, species and host genes with correlation coefficient ≥0.6, and *p*-value ≤ 0.05. Compared with other methods (such as Pearson), the normalized count (gene expression) and component data (relative abundance of microbiota) of Spearman’s correlation analysis performed better (Weiss et al., 2016). Furthermore, to study the host’s response to the species, we used the metascape (Zhou et al., 2019) tool to perform functional analysis on the relevant genes of the species. Finally, the Cytoscape software (version 3.8.2) was used to build the interaction network (Shannon et al., 2003).

## Results

### Mapping the Functional Endometrial Microbiota

**FIGURE 1:**
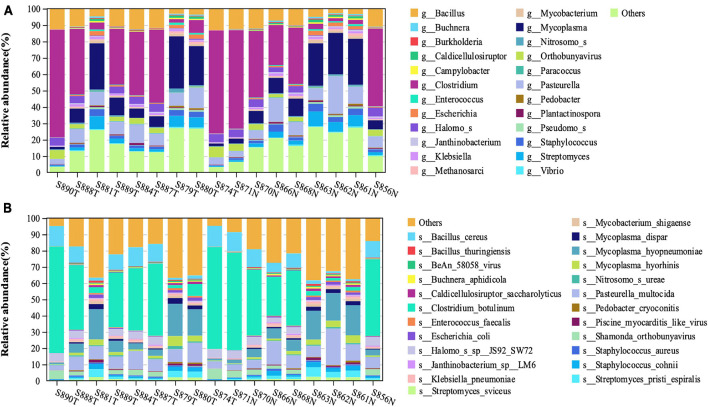
The structural characteristics of the endometrial microbiota. Taxonomic classification of the endometrial microbiota of the EC and normal group at the level of genus **(A)** and species **(B)**.

Through the metatranscriptome analysis pipeline, we identified and analyzed the RNA of microorganisms present in the endometrium. Among the detected RNA sequences, the sequences that failed to be aligned to the human reference genome were about 10%. By using Kraken2 for comparison, a total of 5576 kinds of transcriptionally active *Bacteria* and 381 kinds of transcriptionally active *Archaea* were identified (Supplementary Table 1). Figure 1 shows the top 25 abundance genus (Figure 1A) and species (Figure 1B) in each sample. The top four most abundant bacterial species in the endometrium of EC patients were *Clostridium_botulinum, Mycoplasma_hyopneumoniae, Bacillus_cereus*, and *Pasteurella_multocida.* At the same time, we noticed that the relative abundance of most species is less than 1%, which was consistent with the low abundance of endometrial biomass (Garcia-Grau et al., 2019).

Next, we discussed the species differences in the endometrium between EC and normal groups. Among the transcriptionally active microorganisms detected in endometrial samples, we found significant differences in the abundance of 17 kinds of microorganisms in EC endometrium and normal endometrium (Table 1).

**TABLE 1:** The species identified by metagenomeSeq.

**Species**	**LogFC**	***p*-values**
*Borrelia coriaceae*	1.5971	0.0009
*Mycetocola sp. 449*	1.5590	0.0187
*Anaerostipes hadrus*	1.3859	0.0292
*Altererythrobacter namhicola*	1.2837	0.0100
*Leptospira biflexa*	1.2646	0.0251
*Nitrospirillum amazonense*	1.2608	0.0319
*Modestobacter marinus*	1.2418	0.0420
*Gramella forsetii*	1.2058	0.0194
*Pantoea ananatis*	1.1819	0.0079
*Hymenobacter nivis*	−1.2132	0.0022
*Potamipivirus A*	−1.2217	0.0426
*Acinetobacter equi*	−1.2241	0.0481
*Corynebacterium flavescens*	−1.2801	0.0451
*Streptococcus mitis*	−1.2959	0.0298
*Mycoplasma californicum*	−1.3346	0.0405
*Pannonibacter phragmitetus*	−1.7912	0.0287
*Corynebacterium ureicelerivorans*	−2.0840	0.0039

### Metabolic Pathway Enrichment Analysis Revealed Possible Host–Microbe Interactions

**FIGURE 2:**
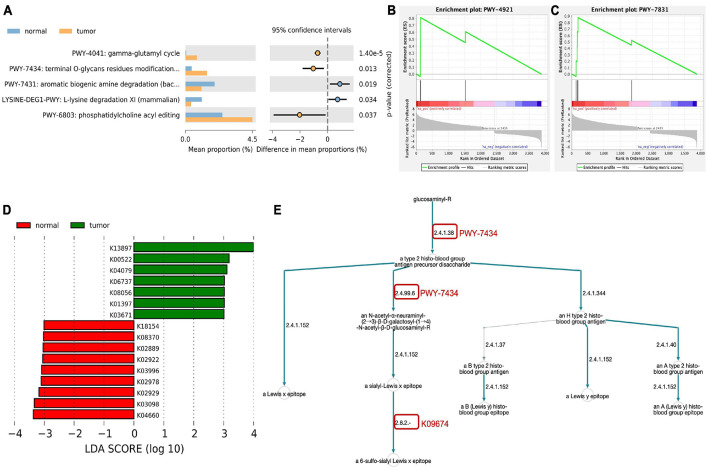
Metabolic pathways enrichment analysis. **(A)** Metabolic pathways of the endometrial microbiota based on MetaCyc database. **(B,C)** MetaCyc-based GSEA functional enrichment analysis of human host. **(D)** Different KEGG orthology (KO) terms between EC and normal group. **(E)** Host–microbial metabolic pathway crosstalk. The red circle indicates the link of microorganisms involved in host metabolic pathway.

To study the possible role of transcriptionally active microorganisms in the endometrium, we analyzed the metabolic pathways of the host and microbiota based on the MetaCyc database. Among the detected microorganisms, three metabolic pathways were significantly enriched in the endometrium of EC patients (Figure 2A and Supplementary Table 2). In the human host, based on the results of DEseq2 analysis, 3885 mRNAs were found to be differentially expressed between the EC group and the normal group (log2-fold-change ≥ | 1|, FDR ≤ 0.05, Supplementary Table 3). The metabolic pathway enrichment analysis of differential genes found that two pathways were significantly enriched, namely, PWY-4921 (protein citrullination, Figure 2B) and PWY-7831 (ABH and Lewis epitopes biosynthesis type 2 precursor disaccharide, Figure 2C). Furthermore, as shown in Figure 2E, we found that the endometrial microbiota of the EC group were involved in multiple links in the metabolic pathway of the host PWY-7831 (marked by the red circle in the figure).

HUMAnN3 was also used for KEGG orthology (KO) enrichment analysis (Supplementary Table 4) and used linear discriminant analysis effect size (LEfSe) (Segata et al., 2011) to screen for differences in KO between the two groups. As a result, we obtained seven KOs that were significantly enriched in the endometrium of the EC group: cystatin-SN (K13897), ferritin heavy chain (K00522), molecular chaperone HtpG (K04079), tumor-associated calcium signal transducer 1 (K06737), protein disulfide isomerase family A, member 3 (K08056), matrix metalloproteinase-7 (matrilysin and uterine) (K01397), and thioredoxin 1 (K03671) (LDA ≥ 3, Figure 2D).

### Integrated Analysis of Interactions Between Host and Microbiota

**FIGURE 3:**
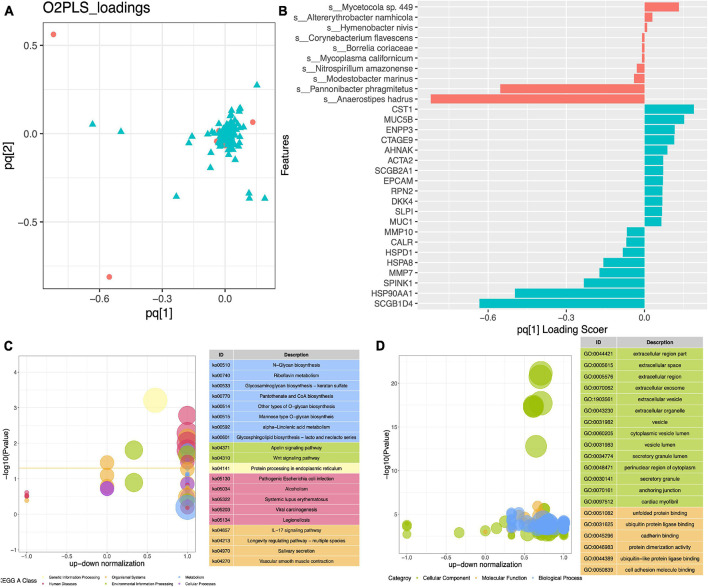
Integrated analysis of interactions between the host and microbiota. **(A)** Loading plot of O2PLS model between differentially abundance species and differentially expression genes. **(B)** Top 10 loading species and top 20 loading genes. KEGG **(C)** and GO **(D)** enrichment analysis of top 50 loading genes.

To further explore the relationship between the activities of endometrial microorganisms and host endometrial gene disorders, we constructed an O2PLS model (Figures 3A,B). Through sevenfold cross-validation, it was determined that the modeling parameters were *n* = 2, *nx* = 1, *ny* = 1, and the MSE = 127.60 at this time. The model’s loading diagram (Figure 3A) and the evaluation parameters of the model (R2X: 0.942, R2Y: 0.519) explain that the model construction is relatively satisfactory. The top 10 loading microorganisms and the top 20 loading genes are displayed in Figure 3B.

We conducted a KEGG enrichment analysis of the top 50 loading genes and found that they were mainly enriched in metabolic-related pathways. In addition, the Wnt signaling pathway, Apelin signaling pathway, and IL-17 signaling pathway were also significantly enriched (Figure 3C). From the results of GO enrichment analysis (Figure 3D), we could see that the main focus was on the functions related to cell adhesion, such as extracellular region part, extracellular space, and extracellular region.

**FIGURE 4:**
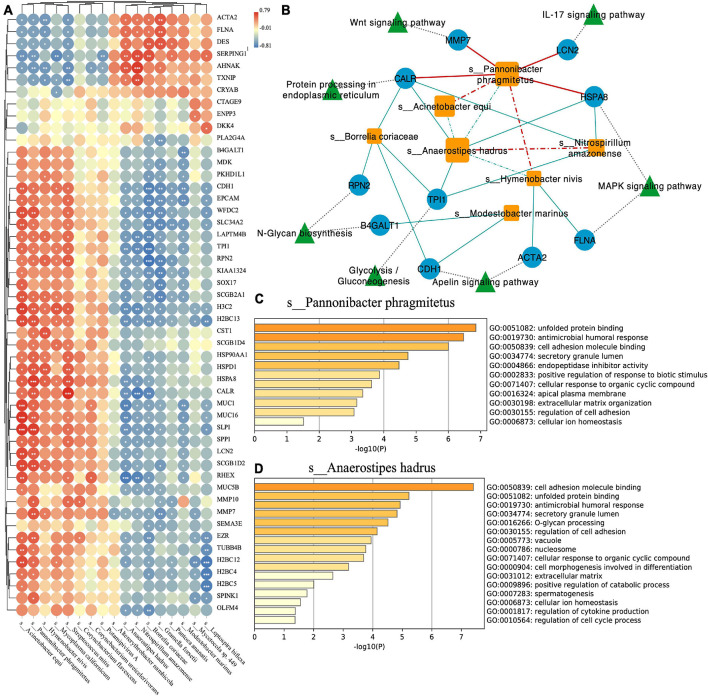
**(A)** Correlation plot depicting top 50 loading genes and top 20 loading species correlations based on Spearman analysis (*** indicates *p*-value < 0.001, ** indicates *p*-value < 0.01, and * indicates *p*-value < 0.05). **(B)** Network visualizing the relationship between species, genes, and pathways. The solid line indicates the positive correlation (red) or negative (blue) relationship between the species and the gene. The dotted line indicates the positive correlation (red) or negative (blue) relationship between the species. Functional enrichment analysis of *Pannonibacter phragmitetus* **(C)** and *Anaerostipes hadrus* **(D)**.

To explore the interaction between species and the host and the relationship between species, we conducted Spearman correlation analysis with the correlation coefficient ≥0.6, *p*-value ≤ 0.05 as threshold (Figure 4A). At the same time, we used Cytoscape software to visualize the network (Figure 4B). We found that *Pannonibacter phragmitetus*, which had a high abundance in EC endometrium, was significantly positively correlated with Wnt signaling pathway, IL-17 signaling pathway, and MAPK signaling pathway (Figure 4B). For the two species with high abundance in the two groups (EC: *Anaerostipes hadrus*, Normal: *P. phragmitetus*), we used the metascape tool (Zhou et al., 2019) to perform functional enrichment analysis on their positively (*P. phragmitetus*, Figure 4C) or negatively (*A. hadrus*, Figure 4D) related genes. We found that the two have standard functions, cell adhesion molecule binding (GO: 0050839). In addition, we also found that the negatively related genes of *A. hadrus* were significantly enriched in other functional items related to cell adhesion, such as extracellular matrix and regulation of cell adhesion (Figure 4C).

## Discussion

For a long time, microorganisms, including bacteria and viruses, have been thought to play an essential role in the occurrence and development of cancer. However, the elucidation of related mechanisms is challenging. Current animal experiments show that the carcinogenic effects of microorganisms may be more related to the overall changes in the microbiome rather than due to a single species (Schwabe and Jobin, 2013).

DNA is a very stable molecule, and unlike RNA that is rapidly degraded, it can exist for decades. Microorganisms are found in almost all body parts, including the endometrium (Glassing et al., 2016), so it is difficult for us to distinguish whether the detected species is active when using DNA-based detection technology for microbial detection.

In this study, our first concern was the composition and structure of functionally active microorganisms in the endometrium of patients with endometrial cancer and the difference between the structures of the microorganisms in the average population. We used the novel metatranscriptomics pipeline to study the functional and active microorganisms of the endometrium of patients with endometrial cancer. The analytical reliability of this novel analysis pipeline had been confirmed in the study of Sola-Leyva et al. (2021). Based on the analysis results, we found that the top five phyla that were significantly enriched in the endometrium of patients with endometrial cancer were *Firmicutes, Proteobacteria, Tenericutes, Actinobacteria*, and *Bacteroidetes* (Supplementary Figure 1). This was consistent with the research results of Walther-Antonio et al. (2016), which once again confirmed the reliability of the novel pipeline used in this study. At the same time, we also got the difference between the two groups of microorganisms. As far as we know, this was the first study to describe the difference in functional species composition at the species level.

Another issue we were concerned about was how these different species interact with the human host. Through enrichment analysis of metabolic pathways based on the MetaCyc database, we found that the microorganisms of EC endometrium participated in the metabolic reaction process of EC endometrium from *N-acetyl-beta-glucosaminyl* to *6-sulfo-sialyl Lewis x epitope* and promoted *6-sulfo -Biosynthesis of sialyl Lewis x epitope*. *6-Sulfo-sialyl Lewis x epitope* was highly expressed in a variety of tumors, and its role was mainly to promote tumor metastasis by adhering tumor cells to blood endothelial cells (Nakagoe et al., 2002). However, it had not been reported in endometrial cancer. *N-acetyl-beta-glucosaminyl* metabolism key enzyme β*-N-acetylglucosaminylglycopeptide*β*-1,4-galactosyltransferase* was closely related to the occurrence of epithelial-to-mesenchymal transition (EMT) in tumors (Zhang et al., 2011; Khan et al., 2018). In our last part of the results, we also found that the highly abundant species in EC had an inhibitory effect on the biosynthesis of N-Glycan. Therefore, we speculated that endometrial microorganisms participate in tumor migration by affecting the metabolic activities of the host’s endometrium.

In addition, by establishing a high-performance O2PLS model, we had further studied the functions of different species. The enrichment analysis results of the top 50 loading genes were roughly the same as the enrichment results of metabolic pathways, such as functions related to tumor migration (*N-glycan* biosynthesis and *Glycosaminoglycan* biosynthesis). At the same time, we found that these species with high abundance in the EC endometrium were closely related to activating the Apelin signaling pathway, which had been confirmed to be related to the increased risk of endometrial cancer (Yang et al., 2016). Another important finding was that species with high abundance in EC had an inhibitory effect on the processing of endoplasmic reticulum proteins. Thus, we speculated that endometrial microorganisms also played an essential role in the unfolded protein response (UPR).

The uterus was a site with very low microbial biomass. In this study, we used a novel analysis pipeline to describe the composition of functionally active microorganisms in the endometrium of endometrial cancer. Exploring the interaction mechanism between these active microorganisms and the host was the focus of our research. As far as we know, this was the first study on the mechanism of microbe–host interaction. We believe that this could provide new ideas for the treatment of endometrial cancer. However, this article still had limitations. The sample size included in this study was small, and the sample size needed to be expanded in the future for more in-depth research.

## Data Availability Statement

The datasets presented in this study can be found in online repositories. The names of the repository/repositories and accession number(s) can be found in the article/Supplementary Material.

## Author Contributions

CF and PC carried out the study. PC and YG analyzed and interpreted the data. TL and TH drafted the manuscript. CF, PC, and TL coordinated the study, participated in the design, and reviewed the manuscript. TL, LJ, JW, and TH took part in revising the manuscript. All authors read and approved the final manuscript.

## Conflict of Interest

The authors declare that the research was conducted in the absence of any commercial or financial relationships that could be construed as a potential conflict of interest.

## Publisher’s Note

All claims expressed in this article are solely those of the authors and do not necessarily represent those of their affiliated organizations, or those of the publisher, the editors and the reviewers. Any product that may be evaluated in this article, or claim that may be made by its manufacturer, is not guaranteed or endorsed by the publisher.

## Supplementary Material

Supplementary Figure 1 Pie chart of taxonomic classification of EC patients at the phylum level.

Supplementary Table 1 Abundance of functionally active microorganisms in all samples.

Supplementary Table 2 Metabolic pathways of functionally active microorganisms.

Supplementary Table 3 Differentially expressed genes between the EC group and the normal group.

Supplementary Table 4 KEGG orthology (KO) enrichment analysis.
