# Supplementary figures and images for: Interaction Between Functionally Activate Endometrial Microbiota and Host Gene Regulation in Endometrial Cancer
Source: Front Cell Dev Biol. 2021 Sep 23;9:727286. doi: 10.3389/fcell.2021.727286 (PMC8495019; doi:10.3389/fcell.2021.727286)

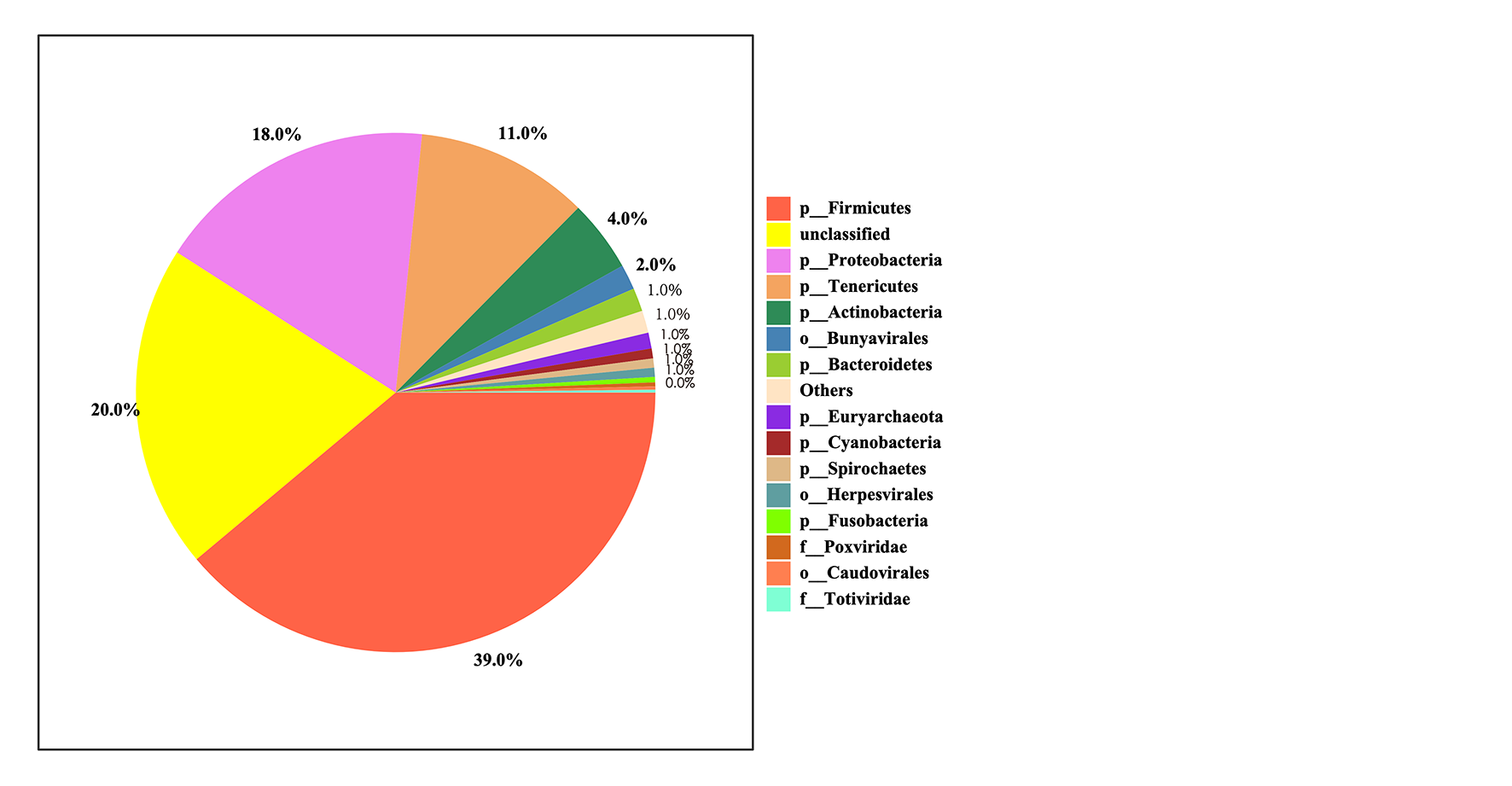

Supplement: Supplementary Figure 1 — Pie chart of taxonomic classification of EC patients at the phylum level. [file Image_1.TIF]
